# Tumor Regression Grades: Can They Influence Rectal Cancer Therapy Decision Tree?

**DOI:** 10.1155/2013/572149

**Published:** 2013-09-25

**Authors:** Marisa D. Santos, Cristina Silva, Anabela Rocha, Eduarda Matos, Carlos Nogueira, Carlos Lopes

**Affiliations:** ^1^Digestive Surgery Service, Department of Surgery, Hospital de Santo António, Largo Professor Abel Salazar, 4099-003 Porto, Portugal; ^2^Department of Community Health, Instituto de Ciências Biomédicas Abel Salazar, Rua Jorge Viterbo Ferreira No. 228, 4050-313 Porto, Portugal; ^3^Pathological Anatomy Service, Department of Pathology, Hospital de Santo António, Largo Professor Abel Salazar, 4099-003 Porto, Portugal; ^4^Department of Pathology and Molecular Immunology, Instituto de Ciências Biomédicas Abel Salazar, Rua Jorge Viterbo Ferreira No. 228, 4050-313 Porto, Portugal

## Abstract

*Background*. Evaluating impact of tumor regression grade in prognosis of patients with locally advanced rectal cancer (LARC). *Materials and Methods*. We identified from our colorectal cancer database 168 patients with LARC who received neoadjuvant therapy followed by complete mesorectum excision surgery between 2003 and 2011: 157 received 5-FU-based chemoradiation (CRT) and 11 short course RT. We excluded 29 patients, the remaining 139 were reassessed for disease recurrence and survival; the slides of surgical specimens were reviewed and classified according to Mandard tumor regression grades (TRG). We compared patients with good response (Mandard TRG1 or TRG2) versus patients with bad response (Mandard TRG3, TRG4, or TRG5). Outcomes evaluated were 5-year overall survival (OS), disease-free survival (DFS), local, distant and mixed recurrence. *Results*. Mean age was 64.2 years, and median followup was 56 months. No statistically significant survival difference was found when comparing patients with Mandard TRG1 versus Mandard TRG2 (*p* = .77). Mandard good responders (TRG1 + 2) have significantly better OS and DFS than Mandard bad responders (TRG3 + 4 + 5) (OS *p* = .013; DFS *p* = .007). *Conclusions*. Mandard good responders had a favorable prognosis. Tumor response (TRG) to neoadjuvant chemoradiation should be taken into account when defining the optimal adjuvant chemotherapy regimen for patients with LARC.

## 1. Introduction

Colorectal cancer is the third most common cancer in developed countries. It ranks second in Portugal, and it is estimated that each year more than 7,000 new cases arise with 8 Portuguese patients with colorectal cancer dying per day, on average [1, 2]. Surgery remains the primary therapeutic tool in the treatment of rectal cancer, and with the advent of mesorectum complete excision (TME) in cancers of the middle and lower rectum, it was possible to reduce the locoregional recurrence [3–6]. However, concerning locally advanced rectal cancer (LARC), this approach has proved insufficient to maintain levels of locoregional recurrence between 4 and 6% [7, 8]. 

Neoadjuvant CRT allows a reduction of regional recurrences, and when there is a complete pathological response (ypCR), an increase in survival is verified [9]. 

The rate of response is better in neoadjuvant CRT compared with long course RT and possibly absent in short course RT with immediate surgery. In fact, the maximal response of the radiation occurs only several weeks after its end [10]. For that reason, surgery has been delayed until 8–12 weeks following neoadjuvant CRT [11–13]. 

The use of neoadjuvant CRT can lead to tumor shrinkage, increases the likelihood of performing a sphincter preserving surgery, and in the surgical specimen increases circumferential and distal margins, with reduction of lymphatic and vascular invasion [14–19]. 

However, the type and remission rate to neoadjuvant CRT remain considerably variable. While some patients may not respond, other patients experience downstaging, and 15–25% have surgical specimens without any viable tumor cells, a condition referred to as pathologic complete response (ypCR) [20, 21]. 

Complete pathological response leads to excellent locoregional management as it provides an increase in survival for stage I values, that is, 90% at 5 years [22–26]. Based on these data, there are centers that in case of a ypCR advocate a policy of “wait and see” reserving surgical resection only for cases of “tumor escape.” The published results of these centers refer to survival rates equal to or greater than those achieved in ypCR patients with resection [27–30]. 

While there are substantial data regarding the relationship between ypCR and improved oncologic outcomes, the prognostic significance of “near complete” response to CRT has not been extensively evaluated [31]. Therefore, the aim of this study was to verify if the association of ypCR with near complete response (good responders) maintains a similar prognostic of ypCR alone in patients with LARC.

To quantify the response to neoadjuvant CRT, different systems can be used which are particularly important in situations where the pathological response is not complete. Most of them have 5 grades, allowing the creation of groups according to the response [20, 32, 33].

This study evaluates the degree of tumor regression according to Mandard classification in patients with LARC who underwent neoadjuvant CRT followed by surgical resection with TME. 

## 2. Material and Methods

A single-institution database was queried for consecutive patients with LARC and biopsy-proven rectal adenocarcinoma who underwent neoadjuvant CRT followed by elective radical surgery with TME with curative intent between January 1, 2003 and December 31, 2011.

Admission criteria were patients with rectal cancers located less than 12 cm tumor distance from anal verge and clinical stage T2N + M0 or cT3/4 N0/+M0. 

Exclusion criteria were patients with other diagnosed neoplasia, short course RT, yp stage IV, R1/R2 surgery, and death during 60 days postoperatively.

All patients receiving neoadjuvant CRT were operated with an average of 8 weeks after the end of radiotherapy and were included in this analysis. The patients receiving short-course radiation were excluded since when immediate surgery is carried out, no downstaging occurs.

Staging assessment included rigid proctoscopy, total colonoscopy, chest, abdominal and pelvic CT scan, endo-rectal ultrasound (ERUS), pelvic magnetic resonance image (MRI) (since 2008), and carcinoembryonic antigen serum levels. 

The neoadjuvant CRT protocol included a total irradiation of 50.4 Gy in 28 fractions and 5-fluorouracil by infusion pump. 

Radical surgery consisted mainly of sphincter saving rectal resection (SSRE) or abdominoperineal resection (APR) with TME. In the operative procedure selection, we considered the distance of the lesion to the anus, the comorbidities of the patient, and the condition of the anal sphincter.

Operated patients were subjected to adjuvant chemotherapy protocol for 6 months performed preferably with 5-fluorouracil (5-FU) or a combination of 5-FU and oxaliplatinum.

Standard pathologic tumor staging of the resected specimen was performed in accordance with the guidelines of the American Joint Committee on Cancer. Circumferential resection margin (CRM) was scored as positive when cancer cells were within 1 mm of the margin. Evidence of ypCR was defined as absence of viable adenocarcinoma in the surgical specimen or the presence of lakes of mucus without tumor cells. The histology of all surgical specimens was reviewed and confirmed by an independent element and was classified based on Mandard tumor regression grade system (Table 1).

We divided our patient population based on TRG Mandard into two groups: good responders defined as Mandard TRG1/TRG2 and bad responders defined as Mandard TRG3/4/5. The two groups were used to evaluate outcome results.

Disease recurrence was evaluated according to location: locoregional (LR), systemic (DR), or mixed. 

None of the patients were lost from followup. 

All surviving patients were observed in our query in the last three months. 

### 2.1. Statistical Analysis

Survival time was defined as the interval between the beginning of neoadjuvant therapy and the date of the last observation.

Oncologic outcomes were evaluated for 5-year overall survival (OS), 5-year disease-free survival (DFS), overall recurrence (OR), local recurrence (LR), and distant recurrence (DR).

Survival curves were performed using the Kaplan-Meier method and compared by log rank test. 

Mandard groups (good/bad) were compared in relation to age, sex, tumor distance from anal verge, clinical stage, surgical procedure performed, and pathological stage (yp-stage) using Student's *t*-test and the *X* ^2^. For survival analysis, the independent variables Mandard TRG, ypN-stage, the ypT-stage, and tumor distance from anal verge were analyzed using Cox's proportional hazard (method forward stepwise). Was considered statistically significant *p* < .05. IBM SPSS Statistics version 20 was used.

## 3. Results

The database query returned 168 patients. We excluded 29 patients: 11 subjected to short course RT, 11 patients with free radial margin ≤1 mm (R1 surgery), 3 patients yp stage IV, and four deaths in 60 days postoperatively. 

### 3.1. Operative Procedure

The surgery performed in 139 patients was a sphincter saving rectal resection, with anastomosis (with or without protective ileostomy) in 88 patients (63.3%). Abdominal-perineal resection was performed in 46 patients, and five patients were subjected to proctectomy with definitive stoma.

The morbidity of the series was 25.11% (Table 2).

### 3.2. Pathology

Stage distribution is shown in Table 3. The average number of dissected lymph nodes in surgical specimen was 8.2 (range 0–22). 

Response to neoadjuvant therapy is characterized in Table 3.

Classification of TRG according to Mandard system allowed us to define two groups as previously mentioned: TRG1 + 2 and TRG3 + 4 + 5. 

We verified a good response to neoadjuvant CRT in 70 patients (ypCR in 25−17.9%) and a bad response in 69 patients (49.6%). 

The two groups of patients (good response Mandard versus bad response Mandard) are statistically comparable in respect to age (*p* = .12), sex (*p* = .52), clinical stage (*p* = .11), and surgical procedures performed (*p* = .13) with the exception of tumor distance from anal verge (*p* = .009), ypN-stage (ypN0/ypN+) (*p* = .001), and ypT-stage (ypT0-2/ypT3-4) (*p* < .001) (Table 4). 

### 3.3. Disease Recurrence

#### 3.3.1. Pelvic Recurrence

Four patients (2.9%) had isolated pelvic recurrence. Considering only the group of patients with a good response, pelvic recurrence appeared in 1 of 70 (1.4%) 45 months after surgery; TRG1 (Table 3).

#### 3.3.2. Distant Recurrence

Distant recurrence without pelvic recurrence appeared in 20 of 139 patients (14.4%). If we consider only patients with a good response, distant recurrence appeared in six of 70 (8.5%) patients (1/25 TRG1 and 5/45 TRG2). For patients who had a complete pathologic response, distant recurrence emerged in one patient (brain metastasis 25 months after surgery).

#### 3.3.3. Mixed Recurrence

Two patients (1.4%) had pelvic and distant disease. Both were classified as bad responders according to Mandard classification.

### 3.4. Survival

The mean followup was 56 months (range 6–125). Five years overall survival (OS) and five years disease-free (DFS) survival were 72.3% and 71.2%, respectively (Table 3).

To the different subsets, survival at 5 years was matched (Table 5).

**Figure 1:**
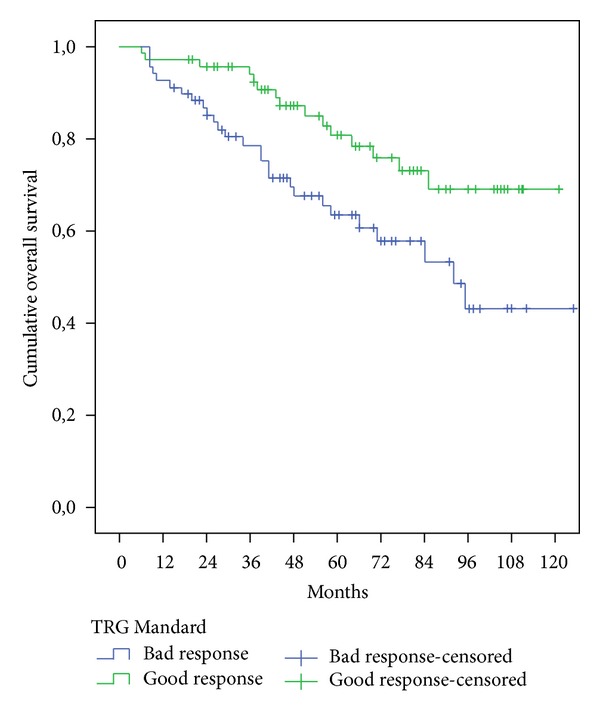
Five years overall survival comparison of the two groups Mandard.

**Figure 2:**
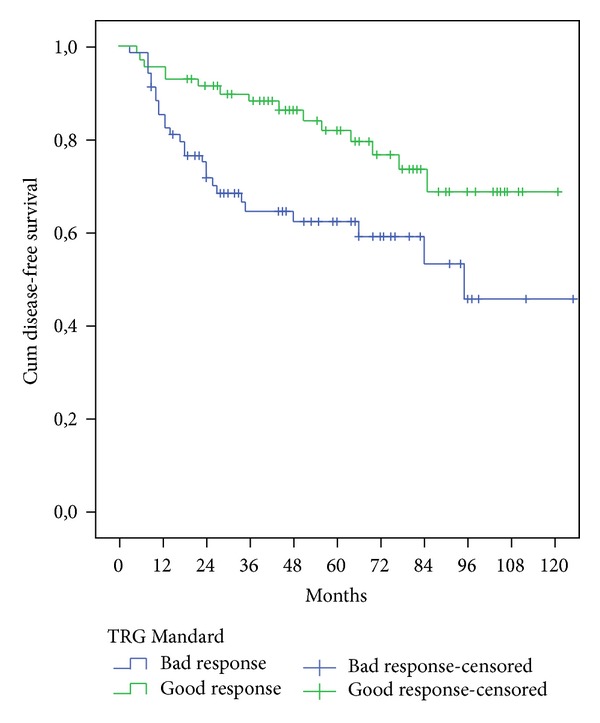
Five years disease-free survival comparison of the two groups Mandard.

The survival of patients who showed a good response on Mandard TGR was significantly higher than the patients with poorer responses in 5-year overall survival (OS) and 5-year disease-free survival (*p* = .013 and  .007, resp.) as we can observe in Table 4 and Figures 1 and 2.

In this series, no statistically significant survival difference was found when comparing patients with complete (ypCR or Mandard TRG1) and partial pathological response (Mandard TRG2) (OS *p* = .77; DFS *p* = .71) (Table 5). 

Overall survival (OS) and DFS in patients with good Mandard response were significantly better than those with a bad response after we enter in the Cox model the following variables: the ypN-stage (ypN0/ypN+), the ypT-stage, and the distance from anal verge (Table 6).

## 4. Discussion

The aim of neoadjuvant CRT in LARC is cytoreduction and downstaging of the tumor, but the tumor response to neoadjuvant CRT is variable. Only when response is good, sphincter preservation rate may increase and reduce the positive radial margin and the positive lymph node in resected specimen (aspects related to rectal cancer prognosis) [17–19, 34, 35]. Prognosis impact of tumor response assessment by TRG is still controversial. Published data are inconclusive [21, 36–42]. Despite uncertain clinical utility of TRG, recently published 7th edition of TNM Staging Manual recommends evaluation of TRG after chemoradiation of rectal cancer as a routine procedure [43]. 

Tumor regression grades evaluate tumor response to neoadjuvant treatment, mainly in CRT. There are several tumor regression systems trying to quantify the response to CRT and ultimately to have a prognostic value [20, 33, 38]. A common, largely accepted, standardized, and validated TRG system does not exist, so the published systems vary in the definition of categories, interfering with studies results.

Mandard TRG was proved effective identifying subgroups with different responses. In our studies, we applied Mandard system, which essentially counts the number of residual tumor cells (Table 1). TRG1 identifies a complete response (ypCR).

The association of tumor response and prognosis has been previously reported. Previous reports have focused on specific T or N downstaging and included in their analysis pCR [44–46]. Other authors have emphasized the value of tumor regression grade, which could more accurately reflect tumor response at a cellular level [32, 33, 47]. In our series, the application of the Mandard system allowed identification of two subgroups of patients with different impact in terms of survival.

We had a complete pathological response in 25 patients out of 139 patients (TRG1). A good response, defined as Mandard 1 and 2 classifications, was present in 70 of 139 (50.4%) patients (Table 3). These percentages are consistent with the available literature [35, 36].

In our series, we did not find a significant survival difference comparing TRG1 with TRG2 (OS *p* = .77; DFS *p* = .71). When we consider TRG1 + TRG2 versus the remaining TRG (3 + 4 + 5), we obtain significant different survival values (*p* = .013). This aspect justified our patients division in two different groups: good responders (TRG1 + 2) and bad responders (TRG3 + 4 + 5). This type of Mandard TRG division was already used by other authors [19, 36, 40]. 

While there are substantial data regarding the relationship between ypCR and improved oncologic outcomes, the prognostic significance of near complete response to neoadjuvant CRT has not been extensively evaluated. In most studies, only the presence of a pathological complete response is correlated to better long outcome and survival improvement [48]. 

Beddy et al. [38] used Mandard TRG and observed better DFS in the combined group of patients having either complete response or near complete response (TRG0 + TRG1) compared with the remaining patients. Dhadda et al. [49] applied Mandard system, and the results obtained suggested improved DFS and OS after preoperative CRT in TRG2 versus TRG3 in the Cox regression analysis. Others series with different TGR system and multivariate analyses failed to demonstrate the prognostic value of TRG for DFS [21, 50]. 

In most studies, the pathologic T category and the nodal status after neoadjuvant CRT still remain the most important independent prognostic factors for DFS [21, 51].

The reason for these different results of the literature can be related to several differences in number of patients of the studies, followup interval, criteria of patients inclusion, regimens of neoadjuvant CRT, time interval between CRT and surgery, R1 definition, TRG system used, and different adjuvant therapy protocols.

Comparing in our study Mandard TRG good response versus Mandard TRG bad response, we find the following:

- Reduction rate of positive lymph nodes −uN+/ypN+ (64.7% versus 36.3%).
- Lower disease recurrence (1.4% versus 7.2% concerning LR; 8,5% versus 21.7% DR; 0% versus 2.8% mixed recurrence).
- Better survival (80.4% versus 63.4% concerning 5-year OS, *p* = .013; 81.7% versus 61.7% 5-year DFS, *p* = .007).

Multivariate analyses confirmed the prognostic value of these two TRG Mandard groups for OS and FDS: OS (*p* = .016); DFS (*p* = .007). 

A good response was associated with an improvement of 54% and 57% in overall survival, and disease-free survival respectively. These results agree with a recent published meta-analysis [31].

Our study is subject to biases and limitations: the series is small, the histology of all surgical specimens was reviewed retrospectively, and the study protocol did not provide extra paraffin blocks from surgical specimen to confirm pCR diagnoses. Another limitation of the present study is the number of dissected lymph nodes: average 8.2 (0–22). The advantages of our study were a single-institution database, patients with LARC treated in the same way, and the histology of all surgical specimens was reviewed and confirmed by an independent and experimented pathologist.

According to data obtained, we identified a subset of patients where the neoadjuvant CRT has the maximum effect and better prognostic (subgroup with higher number of patients than ypCR) and a subgroup of poorer prognosis where other therapeutic regimens will be needed to improved survival.

## 5. Conclusion

Mandard good response (TGR1 + 2) was obtained in 50.4% of our patients with LARC, treated with neoadjuvant CRT and surgery. These patients were associated with lower locoregional recurrence and improved survival compared with Mandard bad response.

Mandard TRG assessment should be, in our opinion, implemented in pathologic evaluation and prospectively validated in further studies.

If Mandard TRG can predict long term outcomes, it can help us to decide when a different adjuvant therapeutic approach is indicated for the patients who undergo preoperative chemoradiation and TME surgery with Mandard bad response (TRG3 + 4 + 5).

## Figures and Tables

**Table 1:** Mandard TRG system.

TRG1	No viable cancer cells, complete response
TRG2	Single cells or small groups of cancer cells
TRG3	Residual cancer outgrown by fibrosis
TGR4	Significant fibrosis outgrown by cancer
TRG5	No fibrosis with extensive residual cancer

**Table 2:** Results—clinical parameters.

Variables	
Sex	
Male	87 (62.6%)
Female	52 (37.4%)
Age	
Mean (range)	64.2 (32–82)
Tumor distance from anal verge	
>6 cm	68 (48.9%)
≤6 cm	71 (51.1%)
Clinical stage	
II	76 (54.7%)
III	63 (45.3%)
Neoadjuvant therapy	
CRT	139
Surgical procedure	
SSRR (sphincter saving rectal resection)	88 (63.3%)
APR (abdominoperineal resection)	46
Other (rectal resection without anastomose)	5
Perioperative complications	
Morbidity	35 (25.1%)
Abdominal or pelvic abscess	11
Anastomose leak	2
Reoperation	5
Readmission	2

**Table 3:** Results—pathological parameters and clinical long term outcome.

Variables	
Postoperative stage	
0	25 (18%)
I	19 (13.7%)
II	53 (38.2%)
III	42 (30.2%)
TRG Mandard	139
Good response (1 or 2)	70 (50.4%)
Bad response (3, 4, or 5)	69 (49.6%)
Overall recurrence disease	26 (18.7%)
Local	4 (2.9%)
Distant	20 (14.4%)
Local and distant	2 (1.4%)
Five years overall survival (os)	72.3% (se = 4.2%)
Five years disease survival (DFS)	72.1% (se = 4.1%)

**Table 4:** Comparison between TRG and demographic and clinic variables.

Variables	TRG1 + TRG2	TRG3 + TRG4 + TRG5	*p* value
Sex			
Male	42 (60%)	45 (65.2%)	.52
Female	28 (40%)	24 (34.7%)	
Age			
Mean (range)	63.1	66.1	.12
Tumor distance from anal verge			
>6 cm	27 (38.5%)	41 (59.4%)	.009
≤6 cm	43 (61.4%)	28 (40.5%)	
Clinical stage			
II	43 (61.4%)	33 (47.8%)	.11
III	27 (38.5%)	36 (52.1%)	
Surgical procedure			
SSRR (sphincter saving rectal resection)	40 (57.1%)	48 (69.5%)	.13
APR (abdominoperineal resection) + other (rectal resection without anastomose)	30 (42.8%)	21 (30.4%)	
Pathological N-stage			
ypN0	61 (87.1%)	36 (52.1%)	.001
ypN+	9 (12.8%)	33 (47.8%)	
Pathological T-stage			
ypT0 − 2	42 (60%)	7 (10.1%)	<.001
ypT3 + 4	28 (40%)	62 (89.9%)	

**Table 5:** Results—TRG and clinical long term outcome. Univariable analysis followup: mean—56 months (range: 6–125).

CRT (*n* = 139)		
Five years overall survival		
Mandard good response (TRG1 + 2)	80.8% (se = 5.3%)	*p* = .013^a^
Mandard bad response (TRG3+ 4 + 5)	63.4% (se = 6.4%)	
Five years overall survival		
ypCR (Mandard TRG1)	80.4% (se = 8.9%)	*p* = .77^a^
Mandard partial response (TRG2)	81.0% (se = 6.7%)	
Five years DFS		
Mandard good response (TRG1 + 2)	81.7% (se = 5.1%)	*p* = .007^a^
Mandard bad response (TRG3 + 4 + 5)	61.7% (se = 6.3%)	
Five years DFS		
ypCR (Mandard TRG1)	80.1% (se = 9.1%)	*p* = .71^a^
Mandard partial response (TRG2)	82.8% (se = 6.1%)	

Se: standard error.

^a^Log rank test.

**Table 6:** Survival in patients TRG (1 + 2) versus TRG (3 + 4 + 5) controlling ypN-stage (ypN0/ypN+), ypT-stage, and the distance from anal verge multivariable analysis.

	Hazard ratio (95% confidence interval)	*p* value
OS	0.46 (0.24–0.86)	.016
DFS	0.43 (0.23–0.81)	.007
